# Gingival Inflammation Associates with Stroke – A Role for Oral Health Personnel in Prevention: A Database Study

**DOI:** 10.1371/journal.pone.0137142

**Published:** 2015-09-25

**Authors:** Birgitta Söder, Jukka H. Meurman, Per-Östen Söder

**Affiliations:** 1 Department of Dental Medicine, Karolinska Institutet, Box 4064, 141 04 Huddinge, Sweden; 2 Department of Oral and Maxillofacial Diseases, University of Helsinki and Helsinki University Hospital, PB 41, FI-00014 Helsinki, Finland; St Michael's Hospital, University of Toronto, CANADA

## Abstract

**Objectives:**

Gingival inflammation is the physiological response to poor oral hygiene. If gingivitis is not resolved the response will become an established lesion.We studied whether gingivitis associates with elevated risk for stroke. The hypothesis was based on the periodontitis–atherosclerosis paradigm.

**Methods:**

In our prospective cohort study from Sweden 1676 randomly selected subjects were followed up from 1985 to 2012. All subjects underwent clinical oral examination and answered a questionnaire assessing background variables such as socio-economic status and pack-years of smoking. Cases with stroke were recorded from the Center of Epidemiology, Swedish National Board of Health and Welfare, Sweden, and classified according to the WHO International Classification of Diseases. Unpaired *t*-test, chi-square tests, and multiple logistic regression analyses were used.

**Results:**

Of the 1676 participants, 39 subjects (2.3%) had been diagnosed with stroke. There were significant differences between the patients with stroke and subjects without in pack-years of smoking (*p* = 0.01), prevalence of gingival inflammation (GI) (*p* = 0.03), and dental calculus (*p* = 0.017). In a multiple regression analysis the association between GI, confounders and stroke, GI showed odds ratio 2.20 (95% confidence interval 1.02–4.74) for stroke.

**Conclusion:**

Our present findings showed that gingival inflammation clearly associated with stroke in this 26-year cohort study. The results emphasize the role of oral health personnel in prevention.

## Introduction

Recently, The Lancet Editorial stated the following: “Any stroke is a terrible event, but a preventable stroke is a tragedy”.[1] The text continues describing the devastating neurological consequences of stroke. According to the World Health Organization statistics, 6.7 million persons died in stroke in 2012 placing it the second leading cause of death in the world, after ischemic heart disease.[2]

Chronic infectious diseases, including gingival inflammation and periodontal disease, have been shown to be involved in the development of cardiovascular diseases and also linked to risk of stroke.[3, 4] In a meta-analysis of cohort studies the risk of stroke did not vary significantly with presence of gingivitis. The review showed nevertheless that periodontitis and tooth loss were associated with the occurrence of stroke. [5]

Gingivitis can develop within days and includes inflammatory changes of the gingiva most commonly induced by accumulation of dental plaque being thus a direct consequence of poor oral hygiene.

Gingivitis and periodontitis are among the most common human chronic infections. It is estimated that 15% to 35% of the adult population in the industrialized countries suffers from these low grade of chronic inflammations.[6] Initial gingival inflammation is the physiological response to oral microbial infection. If this is not resolved the response becomes chronic with subsequently activated adaptive immune response with involvement of cellular and non-cellular mechanisms.[7, 8] Gingivitis often leads to the development of periodontitis with characteristic destruction of the bone surrounding the teeth—and ultimately to tooth loss. [7–9] Gingivitis and periodontitis may last for decades and slowly burden the body by spread of bacteria in the bloodstream and all around the body with subsequent up-regulation of inflammatory mediators.[10] The inflammatory markers are themselves indicators of stroke risk.[11]

Stroke is a major cause of serious long-lasting neurological disability and death also in Sweden where the present study was made.[12] In general, cardiovascular diseases constitute the greatest major health problem in this country where mortality from coronary disease is particularly high.[13] Periodontal disease, in turn, has been shown to associate with heart infarction.[6, 14] Previously our group has shown that young individuals with periodontitis and missing molars, which indicate a history of chronic dental infections, have an increased risk for premature death from diseases of the circulatory system.[15] Furthermore, we have shown earlier that periodontal disease associated with the development of early atherosclerotic carotid lesions.[16]

To this background our current hypothesis was that long-term inflammation of the gingival tissues associates with stroke, the process being part of the chronic oral infection–atherosclerosis paradigm. The specific aim was to study the association between gingivitis and stroke using our longitudinal data covering 26 years.

## Material and Methods

### Study participants, oral clinical examination and questionnaire

The baseline cohort was selected in 1985 using the registry file of all inhabitants (n = 105,798) of the Stockholm metropolitan area born on the 20^th^ of any month from 1945 to 1954 and consisted of a random sample of 3,273 individuals aged 30–40 years. The registry file including all individuals born on the 20^th^ of any month, from 1985 and ongoing, is a unique population file from Sweden. The subjects were informed about the purpose of the study and offered a clinical oral examination in 1985. In total 1,676 individuals (838 men and 838 women) underwent a comprehensive clinical investigation of the oral cavity including, among others, determination of the number teeth, and calculating the dental plaque index (PLI),[17] gingival inflammation index (GI),[18] and calculus index (CLI).[19] Gingivitis was recorded around every tooth using the GI. Background variables such as socioeconomic status, education, regular dental visits and use of tobacco were recorded using a structured questionnaire. Smoking was expressed in pack-years of smoking (number of cigarettes per day multiplied by 365 days and divided by 20 [number of cigarettes in a pack] = the number of packages per year multiplied by the number of years smoked). The original inclusion and exclusion criteria of the patients have been given earlier in our publications.[20, 21]

### Cerebral infarction and socioeconomic data

Data about stroke were obtained from the Center of Epidemiology, Swedish National Board of Health and Welfare, Sweden. The data were classified according to the WHO International Statistical Classification of Diseases and Related Health Problems (ICD-9 and ICD-10). Socioeconomic data were further obtained from the National Statistics Centre, Örebro, Sweden. The data for the incidence of stroke as well as socioeconomic status were obtained from the before mentioned registry files. The data for the incident of stroke in 2011 were compared with the clinical data from 1985.

### Ethical considerations

The study was approved by the Ethics Committee of the Karolinska Institutet and Huddinge University Hospital in Sweden (Dnr 101/85 and revised in 2012/590-32). The study is in accordance with the Declaration of Helsinki.

### Statistical analysis

**Fig 1:**
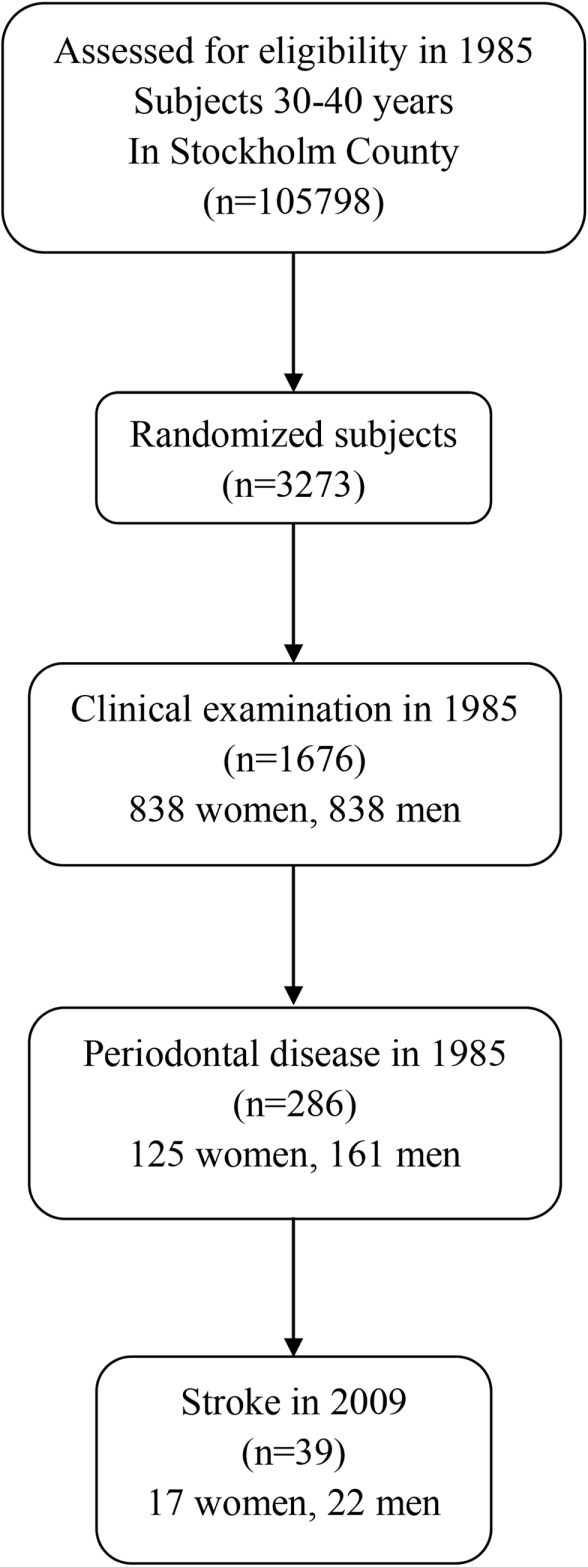
Study profile.

Unpaired *t-*test, chi-square test, and multiple logistic regression analysis were applied when appropriate. We used multiple logistic regression analysis to compare the incidence of stroke according to the state of oral health at baseline, while simultaneously controlling for several potential confounding variables. We included in the model the variables of age, gender, education, income, socioeconomic status, working status, smoking (pack-years of smoking), number of dental visits, scores of dental plaque index, gingival inflammation index, dental calculus index, and periodontal disease record. The outcome variable was the incidence of cerebral infarction. Differences between data sets with a probability of <0.05 were regarded as significant. Two-tailed p-values were used and confidence intervals (CIs) calculated at the 95% level. All statistical analyses were performed using the PASW Statistics software package, version 21 (PASW Inc., Chicago, Illinois, USA). The study profile is shown in **Fig 1**.

## Results

From the study group of 1676 clinically examined subjects in 1985, 39 subjects (2.3%; 22 men, 17 women) got stroke during the 26 years of follow-up. The difference between genders was not significant. Demographic and clinical oral health data at baseline in 1985 of the subjects with and without stroke by the year 2011 are given in Table 1.

**Table 1:** Demographic clinical oral health data of 1676 subjects at baseline examination 1985 with and without stroke 2011.

	Stroke (n = 39) Number, Mean ± SD	No Stroke (n = 1637) Number, Mean ± SD	*p*
Gender, Woman/Men	17/22	821/816	NS
Age, in 2011 (years)	62.7 ± 2.8	61.7±2.9	NS
Smoking, pack-year	5224.7 ± 5404.3	3336.0 ± 4105.8	= 0.01
Education, Compulsery/Higher	11/280	28/1357	NS
Income (Swedish Crowns x 1000)	179.4 ± 10.1	180.1 ± 9.0	NS
Plaque Index	0.74 ± 0.52	0.71 ± 0.49	NS
Gingival inflammaion (GI)	1.46 ± 0.53	1.27 ± 0.53	= 0.03
Calculus index	0.68 ± 0.75	0.45 ± 0.58	= 0.017
Number of missing teeth	1.62 ± 2.12	1.25 ± 2.12	NS

Smoking expressed in pack-years of smoking was significantly higher in the stroke group (p = 0.01) than in those with no stroke. In the group with stroke seven women were smokers, four ex-smokers and six non-smokers, and of men eleven were smokers, four ex-smokers and seven non-smokers, respectively. Gingival index score (GI) and calculus index score (CI) were significantly higher in the stroke group (*p* = 0.03 and *p* = 0.017, respectively). There were no significant differences between the groups with or without stroke regarding age, education, income, dental plaque index score, or in the number of missing teeth. In the multiple logistic regression analysis with stroke as the dependent variable and several independent variables, namely age, gender, pack-years of smoking, education, social and working status, dental plaque, dental calculus and gingival inflammation scores, respectively, GI appeared to be a principal independent predictor associated with 2.20 times the odds of stroke (*p* = 0.044). The results are given in detail in Table 2.

**Table 2:** The results of multiple logistic regression analysis of the relationship between stroke as a dependent variable and several independent variables (age, gender, pack-year smoking, education, social status, working, dental plaque, dental calculus and gingival inflammation.)

Dependent Variable	Explaining Variable	*β*	*X2*	*p-value*	OR (95% CI)
Stroke	Age	1.009	6.66	0.010	2.74 (1.27–5.90)
	Gingival Inflammation,GI	0.789	4.06	0.044	2.20 (1.02–4.74)

Cox & Snell 0.013 square, Nagelkerk 0.66 square

## Discussion

According to the Lancet editorial here earlier mentioned preventing stroke is a major health issue in the world population. In this perspective our present findings indicate that perhaps such simple means as taking care of proper daily oral hygiene might indeed diminish the risk of cerebral infarction. Maintaining good dental health should subsequently be emphasized in all corresponding preventive programs.

This study addressed the issue of statistical association between gingival inflammation and stroke. We observed that the association was significant between clinically recorded gingivitis at baseline and the incidence of stroke 26 years later. Hence, gingival inflammation during decades could indeed be a risk for cerebral infarction later in life. This is a novel finding and shows that not only extended periodontal disease but already gingivitis may pose a threat in this regard.[22] Our results extend earlier findings from case-control studies where an association has been found between cerebral infarction, periodontal disease and gingival inflammation.[5, 23] The particular strength in our study is the long follow-up time, however. In the present study women were diagnosed with cerebral infarction at mean age of 61.9 years and men at 63.4 years, respectively. According to statistics from the Center of Epidemiology, Swedish National Board of Health and Welfare, more than 30,000 individuals got stroke in the year 2012. The mean age was 79 years of the women and 74 years of men, respectively. Thus, in our study the subjects had been diagnosed with cerebral infarction more than 10 years earlier than expected. This observation may further strengthen the importance of early prevention.

The number of stroke patients is closely related to the proportion of elderly in the population because the age-specific incidence of stroke increases steeply by increasing age. The projection of the number of incident stroke patients in Sweden up to year 2050 shows a pronounced effect of the anticipated changes in the population pyramid. Even with stable age-specific incidence of stroke, there will be a dramatic increase of no less than 59% in the number of stroke patients up to the year 2050, whereas the projected increase of the total Swedish population is only 16%. In Europe the population is projected to decrease during the same period, but even so the number of European stroke events is projected to increase up to the year 2025 (despite stable incidence) by approximately 36%. The corresponding figure for Sweden would be 33%. Stroke is therefore a major increasing health problem in many countries.[12, 24]

Regarding the reliability of the present results our subjects were randomly chosen to avoid selection bias. The large subject pool was representative of the ethnically homogenous Swedish adult population, with an age range of 10 years to limit the influence of age differences. The study had a longitudinal prospective design with a cohort of subjects whose oral health status was documented at the baseline 26 years earlier and who had shown signs of gingival inflammation. It can be argued that the gingivitis healed as a result of later visits to a dentist which bias cannot be excluded. A weakness is the lack of data of certain stroke risk factors such as hypertension, diabetes, and dyslipidemia which due to the nature of the study were not available for analyses.

Furthermore, in the present study, we did not include data from oral microbiology of the subjects. Pussinen et al. (2007) have shown that periodontal pathogen *Porphyromonas gingivalis* may particularly associate with stroke by showing in their material that IgA-seropositive men to this bacterium had an OR of 3.31 (1.31–8.40) for stroke.[25] Microbiology of gingivitis, however, is different.[26]

Finally, smoking is known to be one of the important risk factors for cardiovascular diseases as well as for periodontitis.[27] In 2011 in Sweden about one million people still smoked, despite a decline in smoking in the last few decades.[28]Thus it is important to emphasize that in our study population the pack years of smoking were significantly higher in the stroke group compared with subjects with no cerebral infarction.

## Conclusion

In conclusion, the present findings confirmed our study hypothesis by showing a statistically significant association between gingivitis and cerebral infarction. The findings emphasize the importance of maintaining proper daily oral hygiene to reduce the chronic inflammatory burden to the body which, in the worst case, may lead to a catastrophic consequence such as stroke. Maybe oral health personnel should be joined to work together with general practitioners, nurses, and stroke specialists in awareness campaigns for preventing development of stroke.
